# A retrospective analysis of debridement in the treatment of chronic injury of lactating nipples

**DOI:** 10.1038/s41598-021-83172-6

**Published:** 2021-02-11

**Authors:** Haifeng Gao, Jie Wang, Songtao Ding, Yan Li, Yi Zhang, Xiangping He

**Affiliations:** 1 Breast Disease Prevention and Treatment Centre, Haidian Maternal and Child Health Hospital, Haidian District Suzhou Street No. 53, Beijing, 100080 China; 2 Breast Centre, Amcare Women’s and Children’s Hospital, Beijing, China

**Keywords:** Diseases, Health care, Health occupations, Medical research

## Abstract

Treatment strategies for nipple injury are mainly based on aetiology. However, some damaged nipples do not heal after the aetiology was corrected. This study retrospectively analyses the effect of debridement for treating chronic injury of lactating nipples. The medical records on nipple injury management in the authors’ department from December 2015 to January 2020 were retrospectively analysed. A total of 167 patients were enrolled and grouped based on the presence or absence of nipple debridement. The difference in the healing effect, pain relief rate and recurrence rate of nipple injury between the two groups after 1 week was examined. The cure rate of nipple injury in the intervention group (54.3%) was significantly higher than in the control group (26.7%). In addition, the complete pain relief rate in the intervention group (48.1%) was significantly higher than in the control group (23.3%). However, the recurrence rates between the two groups (36.4% (16/44) vs. 34.8% (8/23)) had no statistically significant differences. For patients with no improvement after correction of the aetiology of the in the nipple damage, debridement can improve the healing environment of nipple breakage and thus relieve nipple pain.

## Introduction

The incidence of nipple injury is 58% to 62.9%^1^, which is the most common problem for nursing mothers^2,3^. The pain caused by nipple injury not only brings unpleasant sensations but also subjectively shortens the breastfeeding time^2^. Moreover, it may increase the mental stress of nursing mothers by affecting their mood, sleep and general daily activities, thus influencing milk secretion and objectively shortening the breastfeeding time ^4,5^. Improper or untimely treatment may induce milk deposition and mastitis^6^.

The type and severity of nipple injuries are affected by subjective factors. There is no uniform consesus on the definition, classification and evaluation methods for nipple injuries^7^. Consequently, there is currently no high-quality research on their treatment^8^. Therefore, the evidence-based basis for the treatment of nipple injuries is insufficient.

The principle of nipple injury treatment is etiological and symptomatic treatment. Active management including early detection and treatment would help mothers recover within 2 weeks^9^. Currently, the clinical problem is that some nipple injuries are unresolved after removal of the cause. To evaluate the symptomatic treatment of nipple injuries, Vieira et al*.*^10^ analysed 496 studies on the promotion of nipple injury healing, including the use of lanolin, the combination of lanolin and nipple shields, breast milk, hydrogels, polyethylene film dressings, sprays and distilled water containing chlorhexidine and alcohol. However, these results are uncertain due to the sample size.

In this study, the authors retrospectively selected patients with nipple injury who had no remission after correcting the aetiology and analysed the difference in the treatment effect between patients who had nipple debridement and those without any treatment.

## Materials and methods

### Patients

The medical records of all lactating patients with nipple injuries at the Breast Disease Prevention and Treatment Centre of the Haidian Maternal and Child Health Hospital from December 2015 to January 2020 were collected and analysed. (1) Patients with nipple injuries within 4 months after delivery who presented with white matter keratinisation and necrotic tissue in the nipple, (2) patients with repeated nipple pain, milk deposition, or mastitis in the corresponding area, (3) those whose nipple injuries remained for more than two weeks after the aetiology was corrected, (4) those who have no acute mastitis symptoms such as fever and (5) those whose follow-up period reached 1 month and their information was complete were included in the study. However, (1) patients whose babies showed short lingual frenula or abnormal structures of the palate, (2) those who have nipple infections, (3) those who have flat or concave nipples, (4) those with nipple vasospasmand (5) those who have uncorrected poor breastfeeding methods were excluded.

All patients were divided into the intervention and control groups according to whether nipple debridement was indicated or not.

### Data collection

The following data were retrieved for all patients with nipple injury: demographics, medical history, treatment method, treatment outcomes including the status of nipple breakage after treatment, degree of nipple pain, recurrence and nipple image data.

The healing effect of debridement on nipple injuries was evaluated according to the change in the diameter of the damaged area after treatment. The results were then categorised into the following: cured(complete healing of the injury), improvement(reduction of the diameter)and ineffective(the diameter did not reduce or the diameter increased).

The degree of pain relief was evaluated based on the patients’ pretreatment pain status. The results were categorised into the following: complete remission (pain disappeared), partial remission (pain was relieved) and no remission (no change in pain or pain increased).

To evaluate the recurrence, in patients who were cured, a recurrent lesion in the same site of the same nipple within 1 month was considered a relapse.

This case series was approved by the medical ethics committee of the Haidian Maternal and Child Health Hospital and informed consent for study participation was taken. In addition, all methods were conducted according to the relevant guidelines and regulations.

### Nipple debridement

**Figure1:**
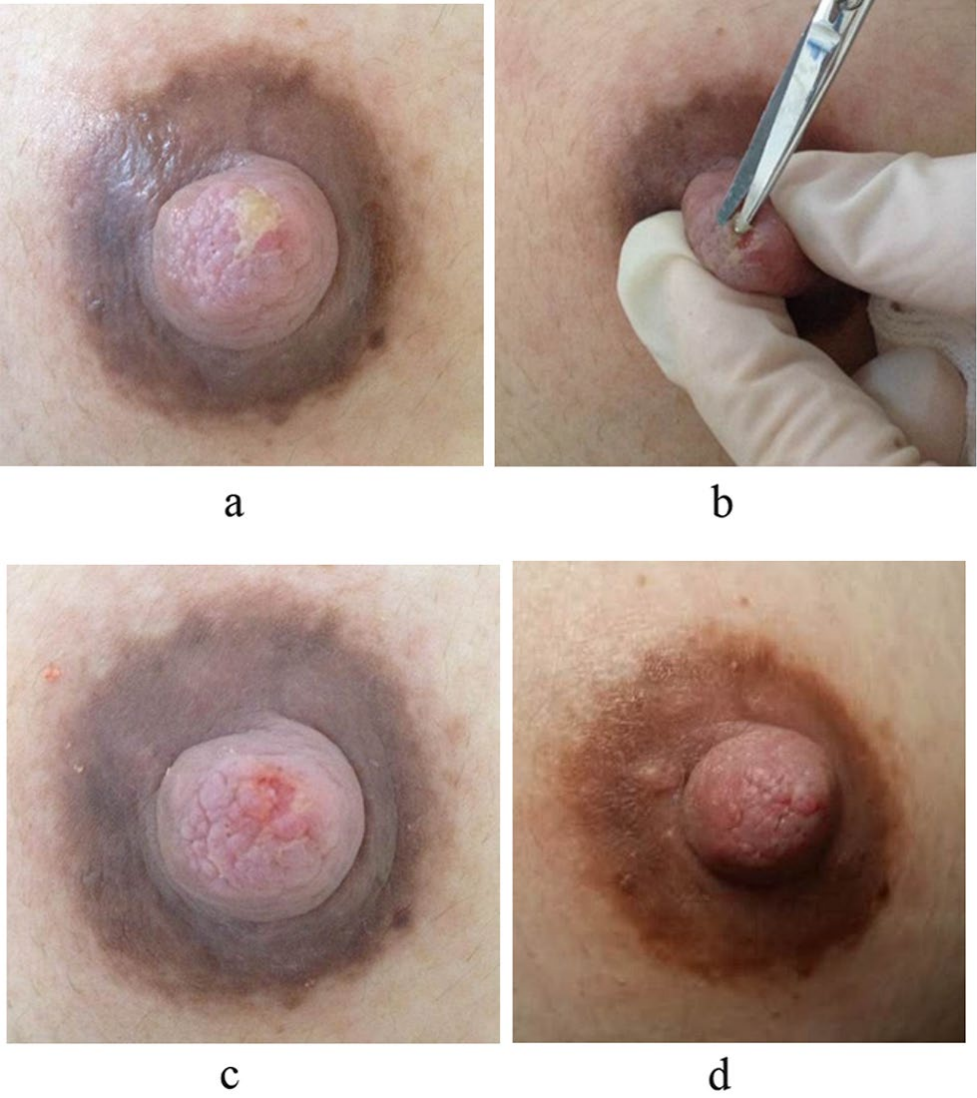
A mother breastfeeding for three months diagnosed with chronic nipple injury. (**a**) Before the treatment, the nipple injury presented as white matter keratinisation and necrotic tissue on the surface of the nipple along with pain. (**b**) The keratinised necrosis tissue was debrided with ophthalmic scissors. (**c**) After the treatment, the necrotic tissue on the surface of the nipple is completely removed and the damaged surface tissue is fresh. (**d**) Five days after treatment, the nipple injury healed completely and the pain was completely relieved.

In the intervention group, the patients underwent nipple debridement. The operation procedure was as follows: the nipple was disinfected by iodophor and anaesthetised by 1% lidocaine. The nipple lesion was completely debrided with ophthalmic scissors to remove the keratinised necrotic tissues until fresh nipple tissue is visible. Nipple debridement is all about the direction and depth to allow the patients to continue to exclusively breastfeed with good sucking after compression and hemostasis (Fig. 1a–d).

In the control group, the patients continued to exclusively breastfeed with good sucking without treatment with medicine or surgery.

### Data analysis

The quantitative variables were expressed in mean, range and standard deviation.

The chi-square test was used to compare the difference in the degree of healing of the nipple injuries, degree of pain relief and recurrence rate between the two groups. The overall significance level was set to an alpha of 0.05. The statistical analysis was carried out using the Statistical Package for the Social Sciences Statistics software version 24.0 (IBM, Chicago, IL, USA).

## Results

### General information and clinical characteristics of the patients

Table1 shows the patients’ demographics and clinical features.

**Table 1:** General information and clinical characteristics of the patients.

General information and clinical characteristics	Intervention group	Control group	p*
Age (years; min–max)	31.14 ± 3.58	31.28 ± 3.31	0.789*
Diagnosis time after delivery (days; min–max)	31.74 ± 25.72	31.49 ± 27.00	0.951*
Total days after the aetiology was corrected (days; min–max)	23.44 ± 5.71	23.19 ± 5.13	0.759*
The long diameter of the damaged lesion (cm; min–max)	0.55 ± 0.17	0.54 ± 0.14	0.542*
Left side (n, %)	35	36	0.601*
Right side (n, %)	46	40	

Values are given as mean ± SD unless otherwise specified.

*P value of < 0.05 was considered statistically significant. Onset time after production.

### Comparison of the treatment effect

The study group consisted of 81 patients, including 44 (54.3%) patients who were cured, 19(23.5%) patients who improved, 18 (22.2%) patients in whom the treatment was ineffective. In the control group consisted of 86 patients, 23(26.7%) patients were cured, 29(33.7%) patients improved, 34(39.5%) patients were in whom the treatment was ineffective (χ^2^ = 13.451;*P* = 0.001) (Table 2).

**Table 2:** Comparison of healing effect of nipple injury.

Group	Cases	Cure	Improvement	Ineffective
Intervention group	81	44 (54.3%)	19 (23.5%)	18 (22.2%)
Control group	86	23 (26.7%)	29 (33.7%)	34 (39.5%)
χ^2^value	13.451			
P*value	0.001*			

*P value < 0.05 was considered statistically significant.

In the intervention group, 39(48.1%) patients obtained complete pain relief, 25(30.9%) patients got partial remission and 17(21.0%) patients had no remission. In the control group, 20(23.3%) patients obtained complete remission, 28(32.6%) patients got partial remission and 38(44.2%) patients had no remission (χ^2^ = 14.170;*P* = 0.001) (Table 3).

**Table 3:** Comparison of nipple pain relief rate.

Group	Cases	Complete remission	Partial remission	No remission
Intervention group	81	39 (48.1%)	25 (30.9%)	17 (21.0%)
Control group	86	20 (23.3%)	28 (32.6%)	38 (44.2%)
χ^2^ value	14.170			
P* value	0.001*			

*P value < 0.05 was considered statistically significant.

### Comparison of the recurrence rates

All cured patients were followed up by telephone 1 month after the treatment. There were 16(36.4%) recurrences in the intervention group and 8(34.8%) recurrences in the control group (χ^2^ = 0.016;*P* = 0.898) (Table 4).

**Table 4:** Comparison of the recurrence rate.

Group	Cases	No recurrence	Recurrence	χ^2^	P*
Intervention group	44	28 (63.6%)	16 (36.4%)	0.016	0.898*
Control group	23	15 (65.2%)	8 (34.8%)		

*P value < 0.05 was considered statistically significant.

## Discussion

After the nipple was damaged, its movement during lactation prevents the creation of a new nipple surface, that is, the chronic wound healing process increased the risk of local infection^11^, and the necrotic tissue in the wound prevented angiogenesis, granulation tissue formation, epidermal regeneration and normal extracellular matrix formation, thus forming a physical barrier for re-epithelialisation, preventing local drugs from directly contacting the wound and affecting the curative effect^12–14^. Anghel et al*.* considered that adequate debridement of necrotic tissues is the core of promoting wound healing ^15^ and a basic step to promote chronic wound healing ^1^. In this study, for patients with no improvement after etiological treatment, nipple debridement was used to remove necrotic tissues. The patients who underwent debridement had significantly better therapeutic outcomes than those untreated with medicine or surgery, suggesting that nipple debridement plays an important role in promoting the healing of chronic nipple injuries. Debridement not only clears the wound but also promotes the growth of fresh epithelial cells, thus speeding the healing process.

In this study, pain relief in the intervention group was significantly better than in the control group. It was shown that nipple debridement can also create favourable conditions for nerve repair, reducing nipple pain. This allows mothers to assess the effect of modifying their breastfeeding methods, to reduce the potential of further injury to the nipple, and to determine and practice methods to achieve and maintain exclusive breastfeeding.

This study has several limitations. First, the retrospective design of this study may have resulted in case selection bias. Second, there was a timing difference between the two groups: nipple debridement in th intervention group was performed starting in the middle of 2017 and the control group was actively observed from 2015 to 2020. Third, there was no strict matching of the causes, and no aetiology was classified and analysed. Fourth, the authors did not compare the frequency and degree of recurrence between the two groups. Last, in this study, the authors defined chronic injury of the lactating nipples as those without relief for more than two weeks after the aetiology was corrected, depending only on the results of subjective individual studies, which need to be discussed further^9^.

In conclusion, this study revealed that nipple debridement on lactating nipples with chronic injury can create good conditions for wound healing, especially for patients with obvious pain and repeated milk deposition. Note that nipple debridement is minimally invasive. It is necessary to minimise damage to normal tissues while removing necrotic tissues to avoid the wound area from increasing and the damage from deepening, thus affecting the function of the mammary duct.

## Data Availability

The datasets generated and analysed during the current study are not publicly available because the study involved the patients’ breasts, which are private body parts, but are available to the corresponding author on a reasonable request.
